# The Work of the Metropolitan Asylums Board.—I

**Published:** 1902-01-04

**Authors:** 


### The Institutional Workshop.

### THE WORK OF THE METROPOLITAN ASYLUMS BOARD.—I.

By the Author of "The Hospital Commissariat."

The voluntary hospitals system of London can at no period be said to have grappled effectually with infectious diseases. They were themselves the sport of infection to a degree which at recurring intervals seriously hampered their work, and their isolation wards were in almost every instance designed for the reception of patients who developed infectious complaints after they had entered the hospital, rather than for the relief of the fever-stricken who came direct from outside.

Two hospitals, it is true, were founded at the commencement of last century for small-pox and fever cases respectively, but the accommodation must even in early days have been wholly inadequate to the needs of the London poor. Typhus was endemic in the thickly populated quarters, and Dr. Lettsom found no better expedient for the fever-stricken than to get them out of their beds and send them to lie on the bridges or wherever they could get the cool air to blow on their bodies. Charitable work was restricted to an extent now hardly credible by the deadly dangers of infection incurred by those who penetrated into these fever dens. It is clear from the literature of the period that the admission of any poor person to private dwellings, or even casual contact in the street, was a subject for just alarm, so impregnated with disease germs were the inhabitants of the slums. The task of purifying London as it then was might well have daunted the reformer.

Take as an example the following extract from "Bleak House," written in 1853 :—

> Between his two conductors, Mr. Snagsby passes along the middle of a villainous street, undrained, unventilated, deep in black mud and corrupt water—though the roads are dry elsewhere—and reeking with such smells and sights that he, who has lived in London all his life, can scarce believe his senses. Branching from this street and its heap of ruins are other streets and courts so infamous that Mr. Snagsby sickens in body and mind, and feels every moment as if he were going deeper down into the infernal gulf.
>
> "Draw off a bit here, Mr. Snagsby," says Bucket, as a kind of shabby palanquin is borne towards them, surrounded by a noisy crowd. "Here's the fever coming up the street!"
>
> As the unseen wretch goes by the crowd, leaving that object of attraction, hovers round the three visitors, like a dream of horrible faces, and fades away up alleys, and into ruins, and behind walls; and with occasional cries and shrill whistles of warning, thenceforth flits about them until they leave the place.
>
> "Are those the fever-houses, Darby?" Mr. Bucket coolly asks, as he turns his bull's-eye on a line of stinking ruins.
>
> Darby replies, "All them are," and, further, that in all, for months and months, the people have been carried out, dead and dying, "like sheep with the rot." Bucket observing to Mr. Snagsby as they go on again that he looks a little poorly, Mr. Snagsby answers that he feels as if he couldn't breathe the dreadful air.

But as the century went on matters grew steadily worse. Various causes contributed to the growth of London, but more by the steady influx to it of persons from all parts of the kingdom than by the normal increase of its own population, scourged as it was by smallpox and fevers of every kind. Great outbreaks of cholera contributed to the general misery and insecurity of large sections of the populace, and at length, tardily, but with ever-rising force, the voice of the sanitary reformer began to be heard. Various powers were from time to time given to local authorities for facilitating the isolation of paupers suffering from infectious complaints. A sanitary Act dealing with the provision of accommodation for persons suffering from infectious diseases was passed in 1866; but this was almost immediately superseded by the Act of 1867, and to the action of Mr. Gathorne Hardy, under which the most remarkable results have been attained during the last thirty years.

It is always impossible to forecast rightly the effect of any comprehensive measure touching the social condition of a large population; the powers conferred by legislation are often employed in a quite unforeseen direction, and the ultimate result is something quite remote from the minds of those who brought about the reform. Of this the Bill of 1867 forms a remarkable illustration.

The Bill arose out of the investigations into the care of the sick in workhouses which were started in 1865 by the *Lancet* newspaper and continued by Government. We shall have occasion again to refer to these investigations, and it is sufficient to say here that they established the fact that the Poor Law authorities had lamentably failed in their duty to the ordinary sick poor of the metropolis, and that they had, with one or two exceptions, entirely neglected to make any separate provision for those suffering from infectious diseases.

The importance of these two branches of the public service varies according to the aspect under which they are regarded. It is true that more individual suffering might be entailed by the total want of skill or even ordinary humanity in the treatment of the chronically sick and aged in the workhouses, but the neglect of the fever-stricken opened up a public danger which had been ignored too long. It appears to have been thought that a double reform was too great a burden to lay on the shoulders of the Poor Law guardians, and in view of the number of unions existing in the metropolis and the difficulty of making separate provision in each for the treatment of infectious diseases and certain forms of lunacy, a general Board for the whole of London was created for the purpose, consisting principally of guardians elected by the various unions. This was the Metropolitan Asylums Board, which was in the first instance an almost exclusively Poor-Law institution for the performance of duties which the individual unions had shown themselves unable to undertake satisfactorily.

The Board was originally composed of 60 managers, 15 of whom were nominated by the Local Government Board and the remainder from the unions and parishes which went to make up the asylums district.

It was enlarged in 1886, and now consists of 18 nominated and 54 elected members.

The responsibilities of the Metropolitan Asylums Board were expressly limited in the order of 1867, under which it was formed, to the care of the "poor persons, chargeable to some union or parish in the said district respectively, who may be infected with or suffering from fever or the disease of smallpox, or may be insane."

The patients with whom they had to deal were essentially and exclusively *paupers*. At the time when the order was framed the respectable poor, however destitute, shrank with horror from the ministrations of the Poor Laws, and there was no reason to believe that any but those driven by absolute necessity would avail themselves of the accommodation to be provided by the managers.

It was not intended that the various parishes should be freed from the burden of maintaining their sick paupers by the constitution of the Board. The cost of maintenance of each patient was borne by the parish or union from which he was admitted, but the whole cost of administration and of erecting suitable buildings was to be defrayed out of a common Metropolitan Fund, for which a rate was levied on each parish, irrespective of the number of patients received.

Such was the constitution of the Metropolitan Asylums Board. Weightier responsibilities have seldom devolved on any new body, and seldom in the history of municipal government have such responsibilities been more faithfully borne.

